# Enhancing the electrochemical performance of TiO_2_ based material using microwave air plasma treatment with an ECR cavity

**DOI:** 10.3389/fchem.2022.1065153

**Published:** 2022-11-24

**Authors:** Ram Swaroop, Pinki Rani, Gaurav Jamwal, Gopikishan Sabavath, Haldhar Kumar, Yogesh Jewariya

**Affiliations:** ^1^ Department of Physics, Central University of Punjab, Bathinda, India; ^2^ Department of Physics Jamia Millia Islamia, New Delhi, India; ^3^ Department of Physics Kandlakoya Medchal, CMR Engineering College, Hyderabad, India; ^4^ Department of Geology, Central University of Punjab, Bathinda, India

**Keywords:** plasma treatments, supercapacitor, cyclic voltammetry (CV), impedance, ECR plasma cavity

## Abstract

The microwave-based plasma treatment facility at the Central University of Punjab Bathinda (CUPB) based on 2.45 GHz has been used to investigate the impact on the electrochemical performance of TiO_2_. This was accomplished by treating a number of pellets of TiO_2_ sample material with microwave plasma at an input power of 80 W. The palette is subjected to microwave plasma treatment at 30-, 60-, 80-, and 100-s intervals. Many such characterization methods, including UV-visible spectroscopy, FTIR, XRD, and FESEM, have been applied to the study of the impact of plasma treatment on other physical and chemical properties in the context of untreated pellets. In the 80-s plasma treatment, the FTIR study showed that the (O-Ti-O) vibration band at 500–900 cm^−1^ was wider than other bands. The UV results showed that an 80-s plasma treatment decreased the sample’s band gap by 37% and increased the amount of disordered, amorphous material in the sample that had not been treated. XRD studies show that a sample that was treated with plasma for 80 s has low crystallinity and a high disorder (amorphous) factor. The Nyquist plot showed that the electrochemical charge transfer resistance drops from 7 (not treated) to 4 after 80 s of plasma treatment. In a study of electrochemical performance, a sample that was treated with plasma for 80 s has a capacitance that is 35% higher than a sample that was not treated.

## 1 Introduction

Due to the exhaustion of nonrenewable energy sources, the world may face numerous energy-related issues in the coming decades. To avoid this energy problem, we need affordable, eco-friendly, and viable renewable energy devices for all people (Tong et al., 2016; Zhang et al., 2016; Ji et al., 2017; Wang et al., 2018; Li et al., 2021; Mu et al., 2021; Cai et al., 2022). Supercapacitors are a prevalent energy storage technology that is utilized in a variety of everyday applications, including electronic devices, automobiles, etc. Supercapacitors had a high power density, a low energy density, fast charge/discharge characteristics, and a charge-discharge life of over 500,000 cycles (Halper and Ellenbogen, 2006). Nonetheless, scientists continue to develop superior supercapacitors capable of delivering high energy densities with increased power density. The superior performance of a supercapacitor is dependent on its electrode material, specifically its porosity. To increase the porosity of the material, the researcher employs various techniques, including composite formation, variation in synthesis technique, doping method, and plasma treatment.

When comparing the aforementioned strategies, the plasma treatment method stands out as the most straightforward and user-friendly for defect engineering (Namdar et al., 2022; Zeng et al., 2022). Plasma treatment modifies the morphology of sample materials faster than conventional techniques such as wet-chemical, hydrothermal, etc., (Garca et al., 1998; Sun and Stylios, 2006; Mao et al., 2017). The plasma treatment with gases generates a distinct functional group along the sample material, which further improves the supercapacitor’s parameters. In addition, plasma treatment redistributes surface electrons and modifies the material’s electric conductivity, which is utilized to its full potential for HER/OER, optoelectronics, and photocatalytic properties (Nakamura et al., 2000; Dinh et al., 2018; Kuriakose et al., 2018). Several of the plasma treatment methods for improving super capacitive performance are discussed in the section that follows.

Literature indicates that microwave plasma treatment of carbon materials in the presence of oxygen, argon, and carbon dioxide gases at a microwave power range of 10–80 W improves supercapacitor performance significantly (Lota et al., 2010; Dulyaseree et al., 2016; Li et al., 2019). This improved performance is attributable to the addition of a different functional group to the electrode material’s surface during plasma treatment. During the microwave plasma treatment of the respective samples, microwave power input, frequency of incident microwave, and pressure condition play crucial roles (Meng et al., 2018). Even under conditions of ambient pressure, plasma treatment can be applied to powder and sample solutions, resulting in an increase in discharge current (Ghanashyam and Jeong, 2020; Sim et al., 2022). For each of these processes, a unique method is adopted for the plasma treatment, or specialized instruments are developed for the purpose (Ghanashyam and Jeong, 2020; Dong et al., 2022; Shin et al., 2022).

In the work being presented here, plasma is treated with the help of an ECR ion source cavity operating at 2.45 GHz TiO_2_ sample material is prepared in conjunction with activated carbon doping. Some pallets are constructed using KBr as a binder. These pallets are subjected to microwave air plasma treatment for varying durations at 100 W of microwave input power. By grinding the pallets into a fine powder and using it as electrode material for a supercapacitor, the pallets are utilized as electrode material. Various characterization techniques are used to examine plasma-treated materials, and cyclic-voltammetry analysis is used to examine the performance of samples treated at various intervals of time. The output outcomes of untreated and plasma-treated results are described in detail in their respective sections of this article. The present report concludes with a comparison to the results of the previous report.

## 2 Experimental description

### 2.1 Plasma treatment facility

**FIGURE 1:**
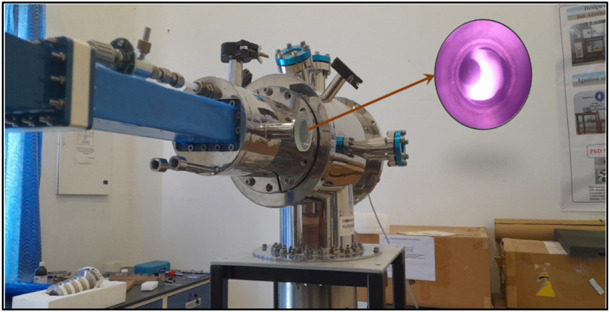
A close view of the 2.45 GHz microwave plasma treatment facility in the absence of permanent magnetic rings.

A 2.45 GHz microwave electron cyclotron resonance facility was designed for ion beam implantation and ECR non-resonance and resonance plasma diagnosis at the central university of Punjab Bathinda. The ECR plasma cavity design is carried so that its radial ports are utilized as multifunctional ports. As previously reported, the ECR plasma cavity can occupy a higher electric field norm inside it (Swaroop et al., 2021). Therefore, non-resonance plasma production is carried out without permanent magnets for the plasma treatment of the materialistic samples. A 1.2 kW magnetron system produces a 2.45 GHz microwave, and a series of waveguide sections transport the microwave from the magnetron to the circular plasma cavity. A close view of plasma diagnosis facilities utilized for the plasma treatment of material samples is shown in Figure 1.

Since the microwave ion source is very sophisticated for transporting the microwave from magnetron to plasma cavity. Here it is also necessary that the cavity becomes the resonance cavity; therefore, any external probe’s presence might disturb the plasma’s production inside the circular cavity. Therefore, to keep in mind the above problem, a Teflon mesh is utilized to hold the sample at the center of the plasma cavity since Teflon is transparent for the microwave.

### 2.2 Material used

Titanium dioxide (TiO_2_, 98%) and potassium hydroxide (KOH, 98%) were purchased from M/s Lobaa Chemie. Activated carbon (AC), Potassium Bromide (KBr, 99%) was purchased from M/s Sigma Aldrich. All samples were used without any further purification.

### 2.3 The synthesis procedure of composite material

**FIGURE 2:**
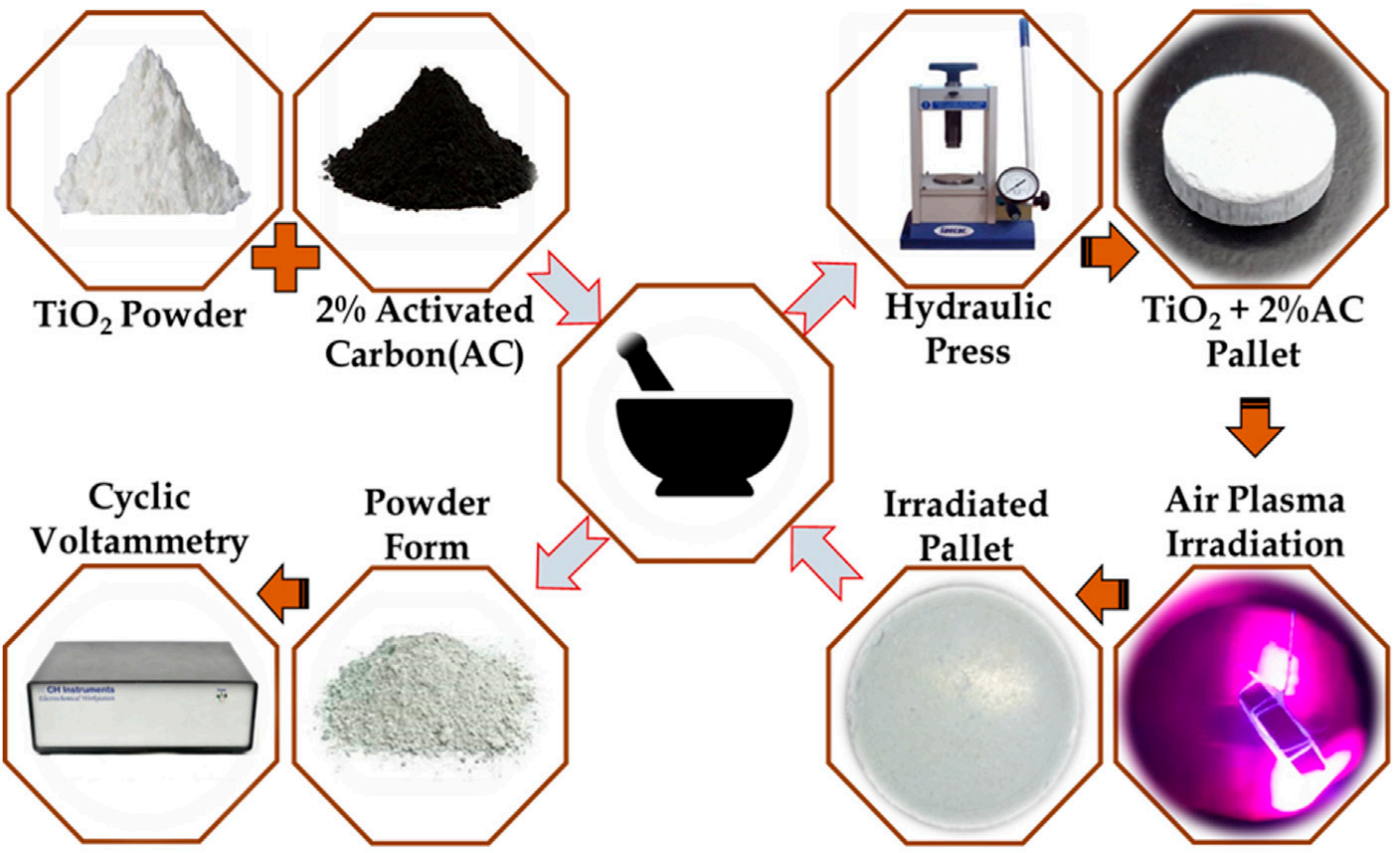
Synthesis methodologies followed for composite TiO2@AC formation (in left above part), pellet preparation procedure for plasma irradiation (in right part), and left below for electrochemical measurements.

A fine powder of TiO_2_ at 2%AC composite sample has been prepared after 1 hour of grinding, and the respective powder form is collected inside the wiles. The prepared sample of ' X ’ mg was taken and added 10% of KBr as a binder. Further, by using a hydraulic pressure unit number of pallets are prepared. A detailed pictorial view of the procedure is shown in Figure 2.

The plasma treatment for the prepared pallets has been carried out at ambient pressure conditions for 30 s, 60 s, 80 s, and 100 s. After the plasma treatment, a respective sample of different treatment times has collected and grinded for fine powder form. The prepared fined powder is an electrode material for cyclic voltammetry study, as shown in Figure 2.

### 2.4 Characterization technique

The electronic properties of the obtained product have been quantified using UV-visible spectroscopy (Shimadzu UV-2450) over 190–900 nm. The vibrational spectroscopic analysis was carried out using Fourier transform infrared spectroscopy (Tensor 27) over 600–4,000 cm^−1^. The study of crystalline phases was carried out using powder x-ray diffraction using (PANalytical X’pert) with Cu K_α_ 1.54 Å X-rays at 2ϴ = 10–90. The surface morphologies were examined using field emission scanning electron microscopy (FESEM, Merlin Compact), including energy-dispersive X-ray spectroscopy (EDS).

### 2.5 Fabrication of electrode assembles for supercapacitor

To study the effect of plasma treatment on super-capacitive performance, plasma untreated and treated sample was utilized for electrode testing. We used circular nickel foam with a radius of 0.4 cm as a current collector. The electrochemical active area of electrode is 0.5024 cm^−2^. The collected samples were mixed with carbon black (12%) and KBr (8%) as a binder substance. A homogeneous slurry was prepared using Methylpyrrolidone (NMP) solution and coated over nickel form and dried at 60°C for 12 h. Here it is noted that the loading mass over elctrode is ∼ 3 mg. A similar process is followed for all the collected samples having different treatment times and kept mass loading constant. For electrochemical studies, a Whatman paper was used as a separator and 6M KOH (potassium hydroxide) as an electrolyte. Then, the electrochemical testing of electrodes was performed using the CHI-760 Electrochemical workstation within two potential ranges −0.5 to 0.5 V and 0 to 1. Here, we measured various parameters such as cyclic voltammetry (CV), galvanostatic charge-discharge, and impedance. All measurements were carried out at room temperature.

## 3 Results and discussion

### 3.1 UV- visible analysis

**FIGURE 3:**
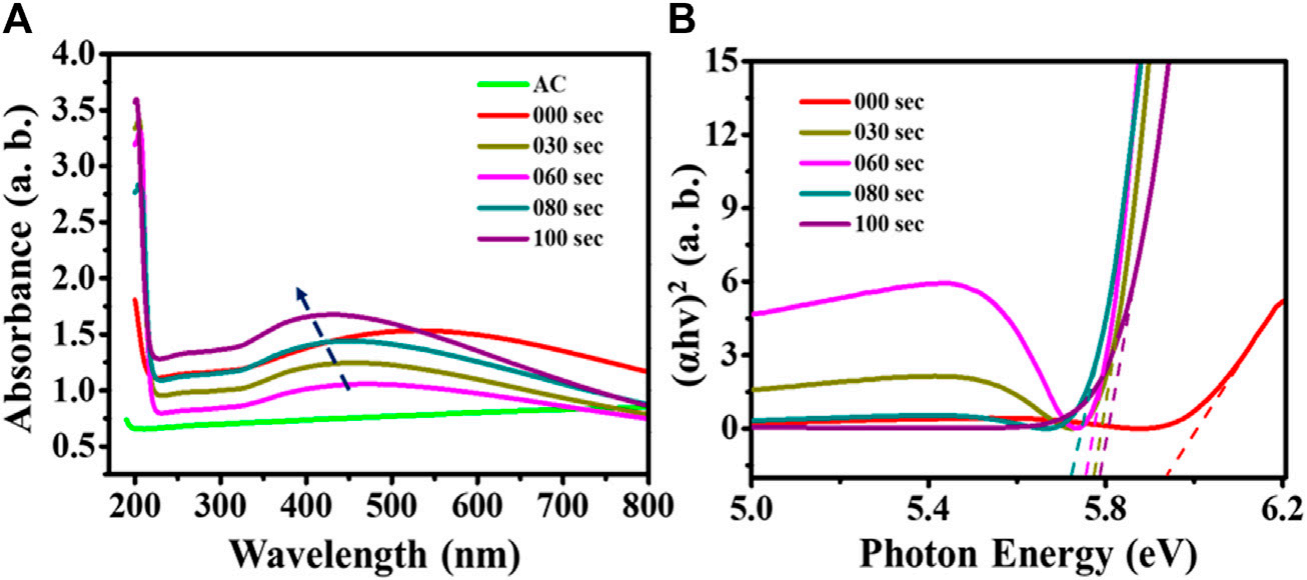
**(A)** UV-Visible spectrum; **(B)** Tauc plot for untreated and microwave plasma-treated samples.

The optical properties analysis of plasma treated and untreated samples were carried out using UV-Visible spectroscopy technique in the range of 200–800 nm. From the UV spectrum, the absorbance peak for activated carbon (AC) emerges approximately at 195 nm. The untreated sample observed two absorbance peaks in the 200 and 400–700 nm range. Here sharp peak at 200 nm is attributed to AC (Qiao et al., 2010), and a broad peak in the range of 400–700 nm corresponds to TiO_2_ transitions (Altaf et al., 2021). On the other hand, in the case of the UV spectrum of plasma treated samples for 30 s, 60 s, 80 s, and 100 s, the broad peak at ∼ 550 nm is shifted towards the lower wavelength region with increasing the plasma treatment time. The blue shift (shift towards low wavelength) in peak shows that the size of the nanoparticle is decreased after plasma treatment, as shown in Figure 3A.

Further, a Tauc equation determines the respective band gap of the untreated and plasma-treated samples, as shown in Figure 3B. From the literature, we found that TiO_2_ has a ∼3.2 eV band gap (Makuła et al., 2018), and potassium bromide has a 7.2 band gap value (Arveson et al., 2018). Figure 3B shows that the untreated samples’ band gap lies below 6 eV (∼5.94), which is higher than TiO_2_ and below from KBr. With the increase in the plasma treatment time, the band gap value of samples is 5.77, 5.75, 5.717, and 5.79 for 30 s, 60 s, 80 s, and 100 s, respectively. This signifies that the 80-s treatment time shows a lower band gap value than other treatment times. Furthermore, a 37.5% decrease in band gap is found from untreated samples compared to the 80-s plasma-treated samples.

### 3.2 Functional group studies

**FIGURE 4:**
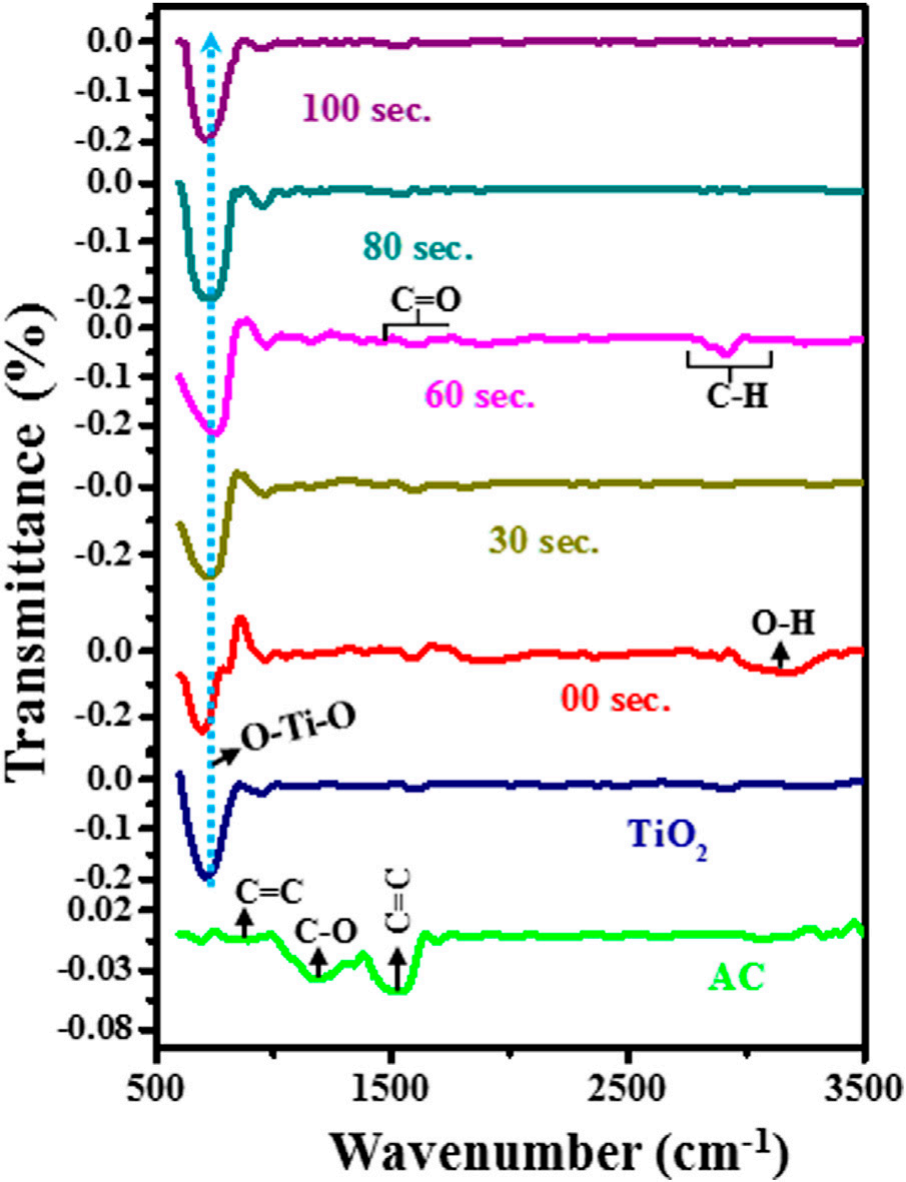
Recorded FTIR spectrum of untreated and treated samples.

FTIR studies have been carried out in the range of 500–3,500 cm^−1^ to characterize the material’s functional group, as shown in Figure 4. The FTIR spectrum of manufactured activated carbon has three vibration bands between 800 and 1700 cm^−1^. The vibration between 750 and 900 cm^−1^ is attributed to C = C bands, the broad vibrations band between the 900–1,300 cm^−1^ corresponds to C-O bands, and 1,500–1700 cm^−1^ shows the C = C group bands. All peaks are well matched with the previous literature (Tran et al., 2017). TiO_2_ has a sharp vibration band of ∼500–900 cm^−1^, which is attributed to the bending vibration of O-Ti-O bands. Also, some small vibration bands appear in the range of 1,100–1,400 cm^−1^, which are assigned to the C-O vibration bond. The slight vibration band between 2,800 and 3,000 shows the stretching band of the C-H bond. The small intensity of hydroxyl peak ∼3,200 cm^−1^ shows little moisture absorbed by the TiO_2_ powder sample. All peaks are well matched with the reported literature (Praveen et al., 2014).

Further, the FTIR spectrum of untreated composite material of 2%AC at TiO_2_ with 10 wt% KBr binder. It was observed that due to the small doping of AC into TiO_2_, the intensity of the vibration band for activation carbon is very low compared to the intensity of TiO_2_ bands. In the untreated composite sample, the intensity of hydroxyl vibration is increased because of the presence of KBr, which easily captures the moisture at room temperature. In the FTIR spectrum of the plasma treated sample, we observed that the vibration band of O-Ti-O is shifted towards high wavenumber and symmetric nature of peak change to asymmetric. It suggested that the symmetry between O-Ti-O bonds exists in the untreated sample. However, as we increase the plasma treatment time, asymmetric stretching occurs in the O-Ti and Ti-O groups of O-Ti-O bonds.

This asymmetric nature is favorable for enhancing electrochemical performance (Zeng et al., 2011). Moreover, we noted that up to a 60-s plasma treatment sample has a sharp vibration band of 500–900 cm^−1^, but for an 80-s plasma treatment sample, this vibration became wider, and after that, for 100 s, we again saw less broaden vibration band. This suggested that in the 80-s sample, there exists a high order of disorder than in another sample. Along this, with plasma treatment, we found that the intensity of the C-O band at 1,100–1,400 cm^−1^ is decreased. Moreover, a new peak appears in the range 1,650–1750 cm^−1,^ attributed to C = O bands. Further, at ∼ 2,800 cm^−1^ wavenumber, the intensity of the CH band firstly decreases at 30 s and again increases with increased plasma treatment time. This shows that there is stretching between the CH bands during plasma treatment. However, the intensity of the hydroxyl peak is decreased with plasma treatment.

### 3.3 Cystography analysis

**FIGURE 5:**
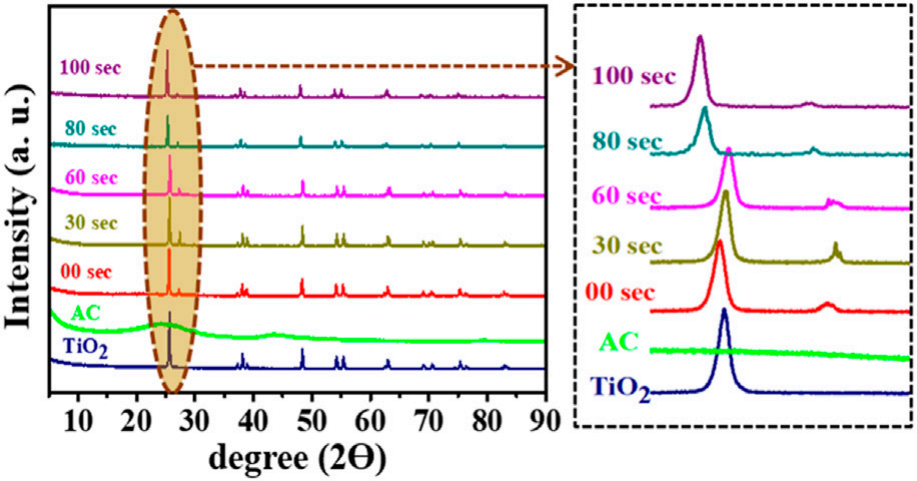
Recorded XRD graphs for untreated and microwave plasma treated samples.

The crystal structure of untreated and plasma-treated composite samples has been investigated using the X-ray diffractometry technique, and corresponding XRD results are shown in Figure 5. The XRD pattern for AC is well-matched with the literature (Xie et al., 2014), and all XRD diffraction peaks for TiO_2_ was well-matched with the three different X’pert high score pdf reference number: 01-089-5010, 00-002-0406, 01-073-1764. This reference pattern shows that TiO_2_ powder has two types of crystal structure; one is tetragonal with lattice parameter: a=b=3.776 Å and c=9.486 Å, and the other is cubic with lattice a=b=c=4.17 Å. The percentage of Tetragonal structure dominates over cubic structure and found the bare TiO_2_ has anatase phase. In the case of the untreated sample, the presence of a low quantity of activated carbon has no significant effect on the XRD patterns, i.e., crystal structure. It signifies that the XRD pattern of the untreated sample is almost similar to that of pristine TiO_2_; in addition, different peaks appear at ∼ 27.5°, which contributes to the presence of KBr. XRD of KBr was well-matched with the X’pert high score pdf reference number 00-001-0695 and showed a cubic structure ( a=b=c=3.285 Å) of KBr is involved.

On the other hand, as we go towards the XRD pattern of the 30 s to 60 s, all peaks are slightly shifted to higher two ϴ values, as represented in the zoomed portion. The peak at ∼ 27° split into doublet peak in which one is shown for cubic KBr presence, and another small peak shows the rutile TiO_2,_ which matched with X’pert high score pdf reference number 01-076-0649. Moreover, with plasma treatment, the overall structure of TiO_2_ remains a combination of tetragonal and cubic structures. Further, as we go towards the XRD patterns of the 80-s plasma treatment sample, the anatase phase obtains for TiO_2,_ which has lattice parameter of the order, a=b=3.783 Å & c=9.51 Å, and KBr has a cubic structure with a=b=c=6.578 Å . For 100-s plasma treatment, lattice papermaker in the tetragonal structure becomes =b=3.789 Å & c=9.54 Å . From the 80 s and 100 s, XRD peaks are shifted to lower 2ϴ, indicating the extension in the lattice parameter values. Compared to the untreated sample, we observed that with plasma treatment, there is a change in bond length of TiO_2_ and KBr material, but the overall crystal structure remains the same, i.e., tetragonal + cubic.

Further, the degree of crystallinity of untreated and plasma-treated samples has been determined using the relation:

$$\%\;crystallinity=\frac{Area\;under\;the\;intense\;peak}{total\;area\;the\;curve}\times 100$$

**FIGURE 6:**
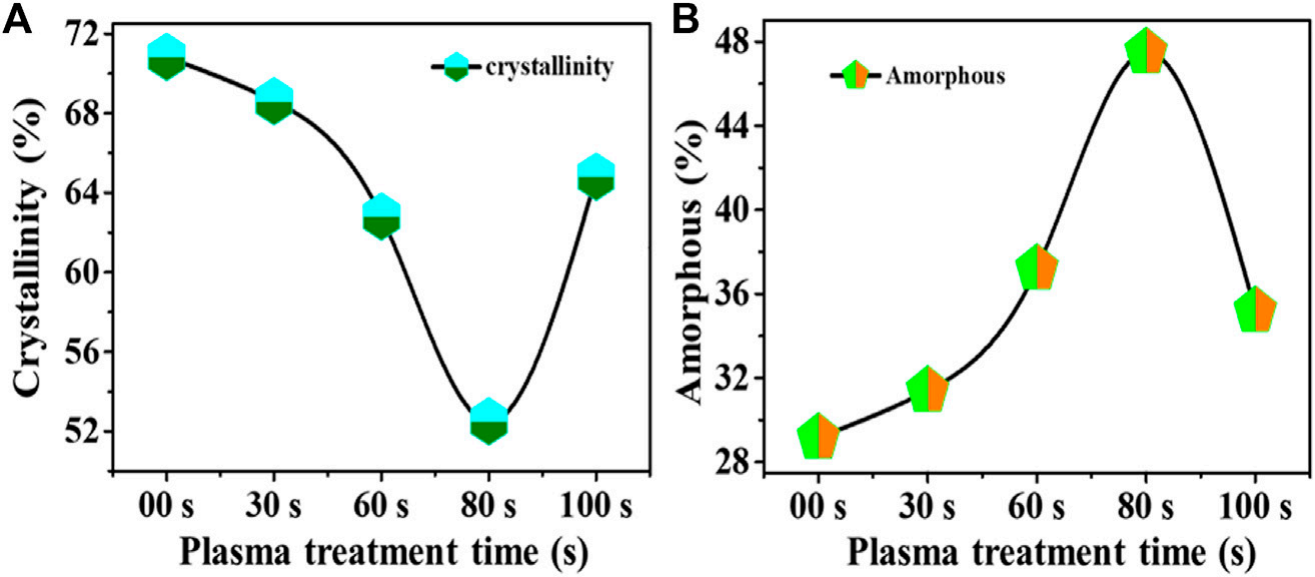
Determined: **(A)** Crystallinity, **(B)** Amorphous percentage in untreated and plasma treated samples.

The determined crystallinity is shown in Figure 6A, where the untreated sample has a higher degree of crystallinity than 70%. With increasing the plasma treatment, crystallinity of the sample first decreases like 68%, 62%, and 52% for 30 s, 60 s, and 80 s plasma treated, respectively, then crystallinity factor increases for 100 s up to 64%. Furthermore, the percentage degree of amorphous has been evaluated, as shown in Figure 6B. It was found that the 80-s sample had a higher degree of amorphous (∼50%) than untreated and other plasma treatment samples.

### 3.4 Morphology studies

**FIGURE 7:**
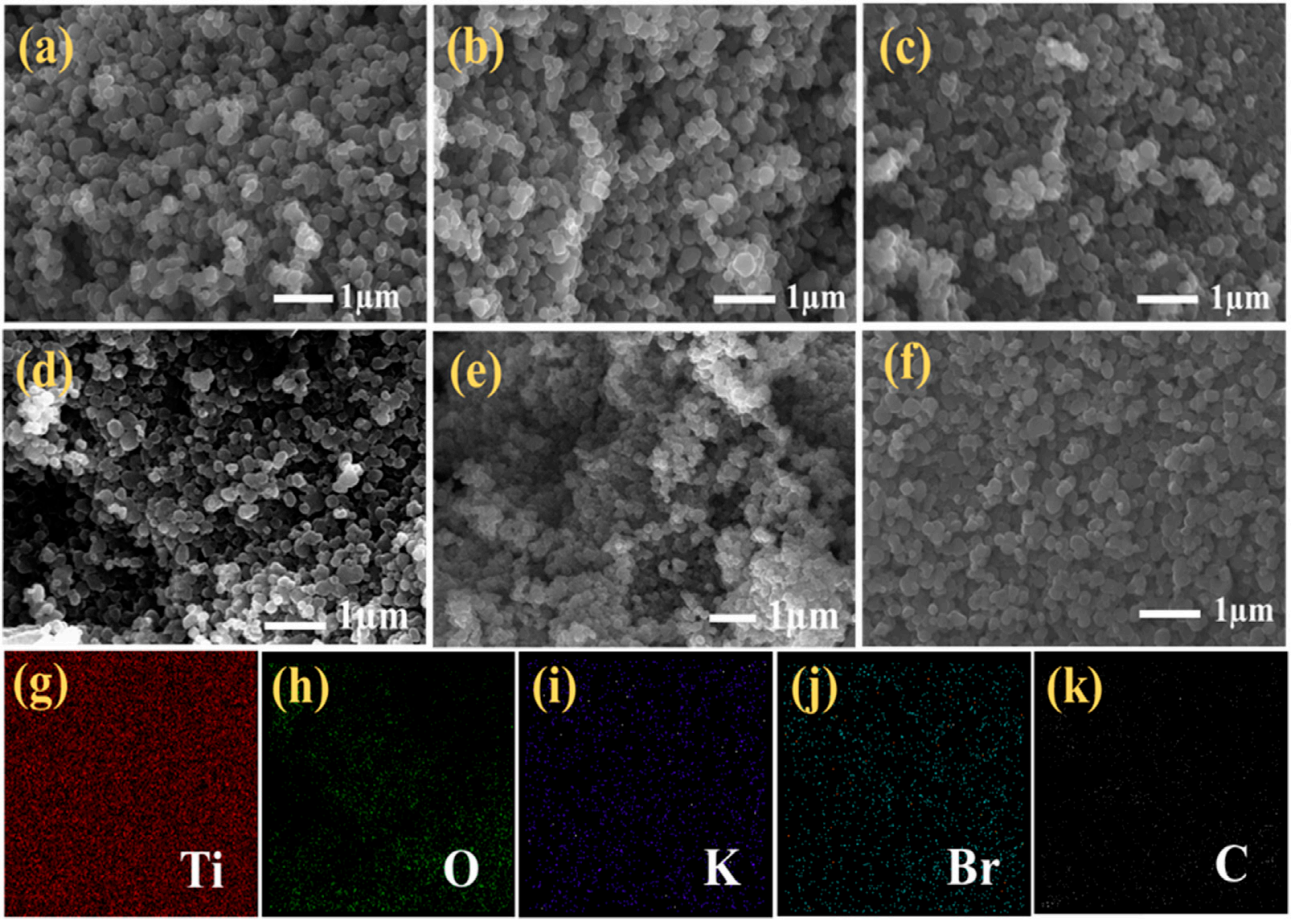
FESEM image of **(A)** untreated sample; **(B–F)** microwave plasma treatment for 30 s, 60 s, 80 s, 100 s, and 120 s; **(G–K)** Elemental mapping of 80-s plasma treatment sample: Ti, O, K, Br, and C.

To examine the effect of plasma treatment, the surface morphology of the sample material has been determined with the help of the FESEM technique with a working distance of ∼5.5 nm at an operating voltage of 20 kV. Figure 7A shows the FESEM images of the untreated sample. Figures 7B–F shows the FESEM images of microwave plasma treatment samples for 30 s, 60 s, 80 s, 100 s, and 120 s, respectively. A nanoparticle formation has been observed in all collectively FESEM images, although no specific change is honored when we simultaneously compare it with any next upcoming treatment time.

However, if we compare the untreated and 120-s microwave plasma treated samples, it is observed that with an increase in the plasma treatment time, the degree of agglomeration is decreased, and nanoparticles become dispersed over the surface. However, there is no change in morphological shape. It is also hard to detect the typical roughness of the untreated and treated samples. The energy dispersive X-ray elemental mapping for the 80-s plasma treated sample is shown in Figures 7G–K. It confirms the elemental composition are Ti, O, K, Br, and C and shows the uniform distribution of all elements over a carbon tape substrate.

### 3.5 Electrochemical performance in −0.5 to 0.5 potential window

**FIGURE 8:**
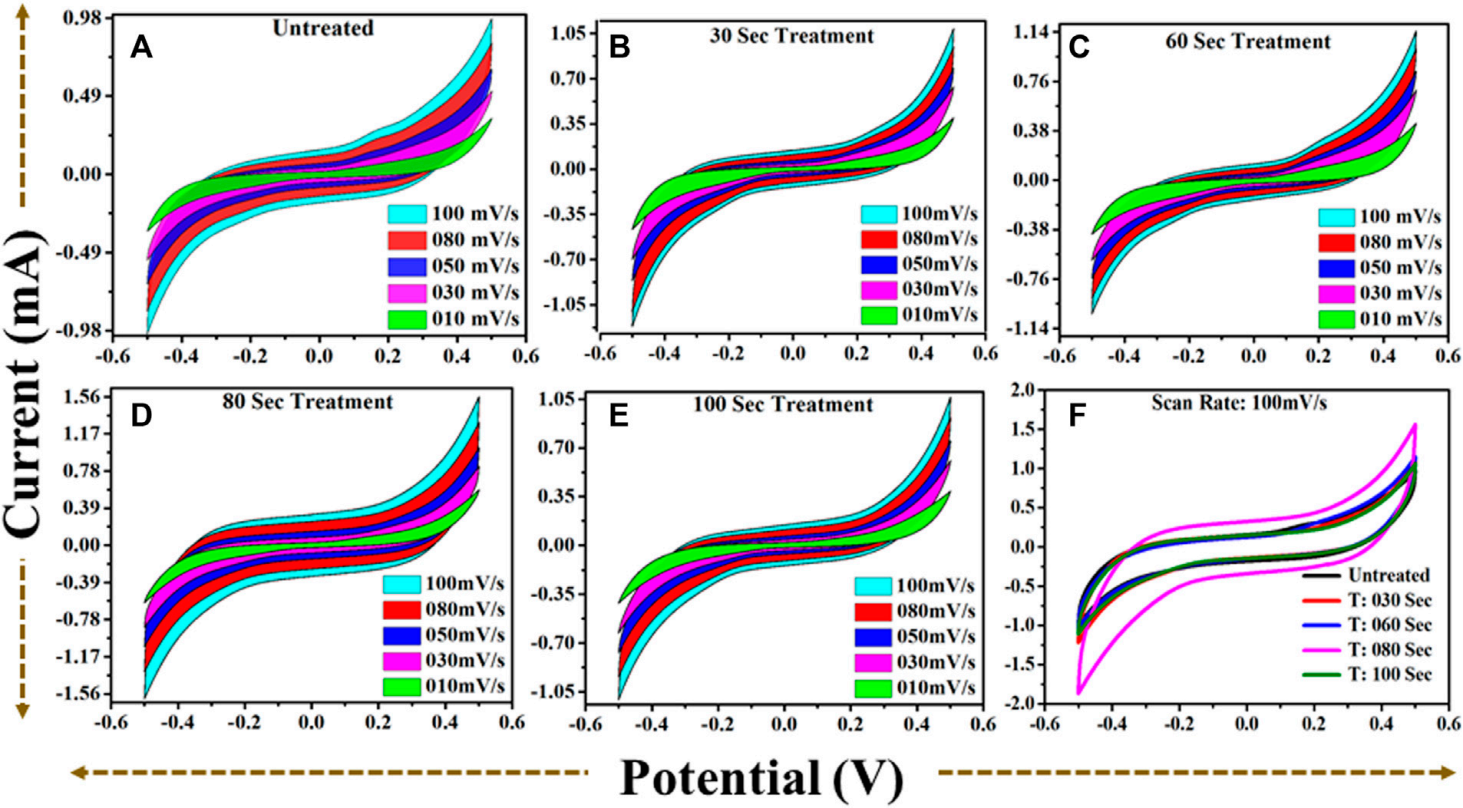
**(A)**: CV profile for untreated TiO2 @scan rate (10–100) mV/s. **(B)**: CV profile for 30 s plasma treated TiO2 @scan rate (10–100) mV/s. **(C)**: CV profile for 60 s plasma treated TiO2 @scan rate (10–100) mV/s. **(D)**: CV profile for 80 s plasma treated TiO2 @scan rate (10–100) mV/s. **(E)**: CV profile for 100 s plasma treated TiO2 @scan rate (10–100) mV/s. **(F)**: A comparative CV profiles for **(A–E)** at scan rate 100) mV/s.

The electrodes were prepared for the untreated, and microwave plasma treated samples to perform the electrochemical study. The present study is conducted for the two electrode configurations in a 6M KOH aqueous solution. Detailed cyclic-voltammetric (CV) profiles for the two-electrode system were obtained at different scan rates from 10 mV/s to 100 mV/s in a −0.5 V–0.5 V potential window, as shown in Figure 8.

**FIGURE 9:**
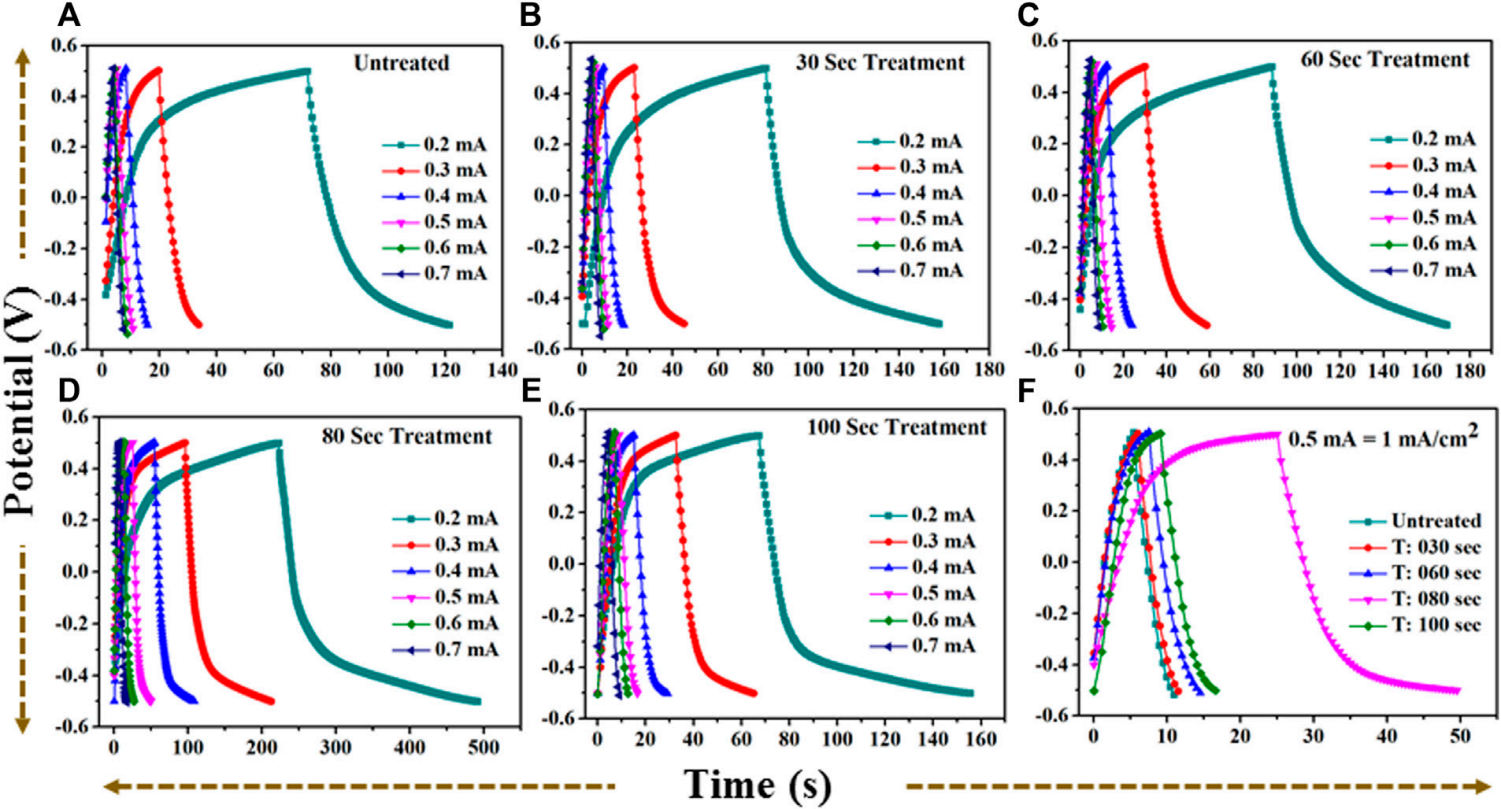
**(A)**: GCD profile for untreated TiO2 at current (0.2–0.7) mA. **(B)**: GCD profile for 30 s plasma treated TiO2 at current (0.2–0.7) mA. **(C)**: GCD profile for 60 s plasma treated TiO2 at current (0.2–0.7) mA. **(D)**: GCD profile for 80 s plasma treated TiO2 at current (0.2–0.7) mA. **(E)**: GCD profile for 100 s plasma treated TiO2 at current (0.2–0.7) mA. **(F)**: A comparative GCD profile for **(A–E)** at current 0.5 mA = 1 mA/cm2 current density.

From all collective CV profiles, it is observed that the area under the curve shows a gradual change for the microwave plasma treated samples having a period: of 30, 60, 80, and 100 s compared to the untreated TiO_2_. The critical area under the curve for untreated and microwave plasma treated samples in different colors is in Figure 8. It is also found that microwave plasma treatment time at 80 s has a higher critical area under the curve than the comparison to the other sample at different plasma treatment times (0 s, 30 s, 60 s, and 100 s). This implies that 80 s microwave plasma treated sample has better capacitive performance than the other samples. Similarly, the respective plots are also obtained for the galvanostatic charge-discharge (GCD), as shown in Figure 9. GCD were performed at different current density from 0.2 mA/cm^2^ to 1.4 mA/cm^2^. It is observed that the charging and discharging time decreased with the increase in current density. As we know from the literature, the longer the discharging time, the higher the electrode’s capacitance will be. A comparative study of the GCD curve at 1 mA/cm^2^ current density for all samples is shown in Figure 9F.

The discharge time for untreated and treated samples are 5.49 s, 5.54 s, 7 s, 24.45 s, and 7.42 s for 0 s, the 30 s, 60 s, 80 s, and 100 s microwave plasma treatment, respectively. At 1 mA/cm^2^ current density, the electrode-potential drop (IR drop) for 0 s, 30 s, 60 s, 80 s, and 100 s treated sample are 171 mV, 181 mV, 162 mV, 104 mV, and 154 mV, respectively.

At 80 s, the plasma-treated sample possesses excellent symmetry and has a higher discharging time and minimum IR drop, which is beneficial for higher capacitance than other treated samples. Furthermore, electrochemical activity has been evaluated using CV and GCD by determining the specific capacitance. The specific capacitance/areal capacitance (C_A_) from the CV plot is determined by relation (Wang et al., 2012):

$$C_{A}=\frac{Area\;under\;the\;curve}{v*∆V*S}$$

here, ' ∆V ' is the potential window measured in volt, “ v ” is the scan rate measured in “mV/s,” and “S” is the surface area of the working electrode measured in cm^2^. The specific capacitance from the GCD curve is calculated using the formula (Wang et al., 2012):

$$C_{A}=\frac{I*∆t}{∆V*S}$$

**FIGURE 10:**
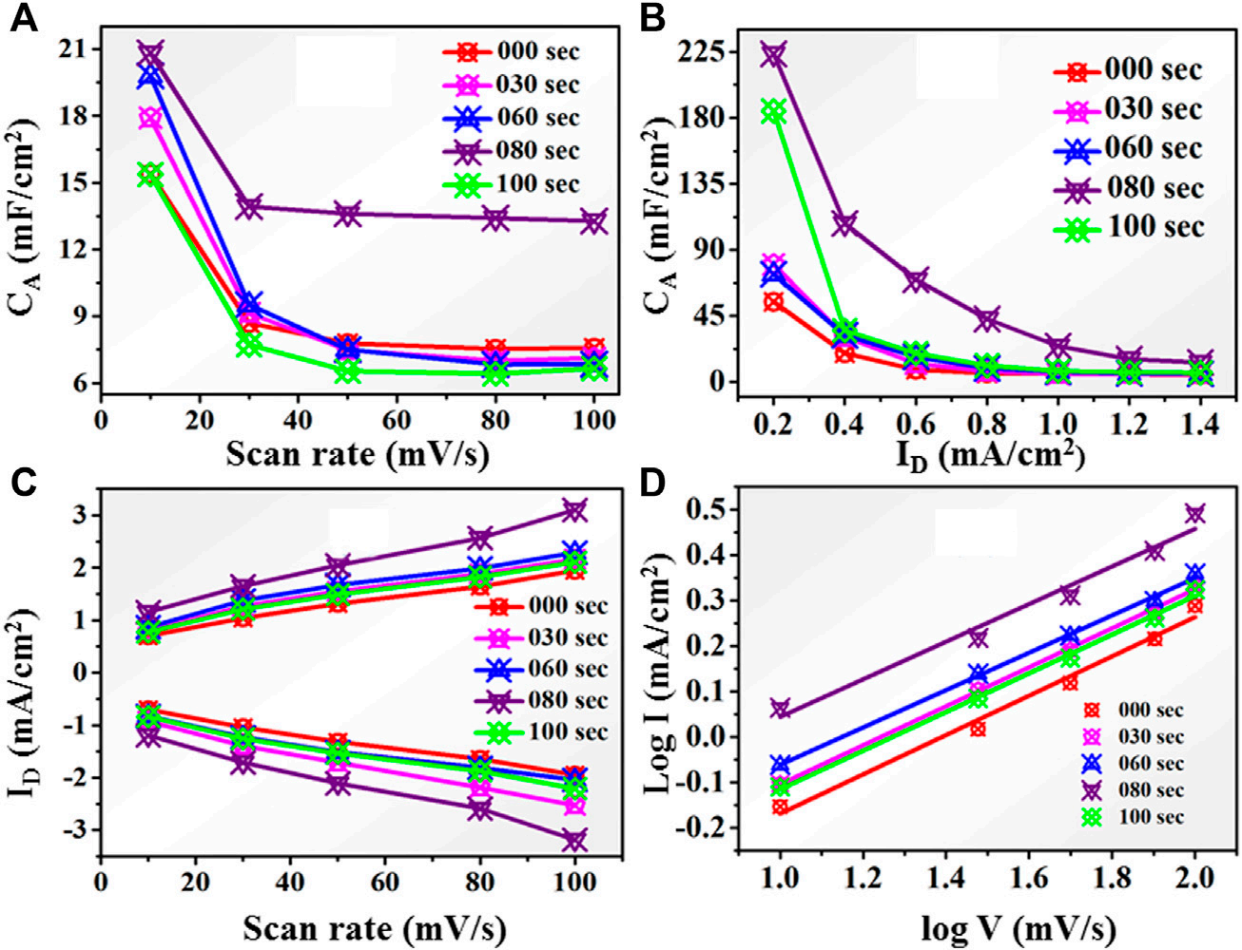
**(A)** Areal capacitance behavior for untreated and 30, 60, 80, and 100-s microwave plasma-treated samples at different scan rates. **(B)**: Areal capacitance behavior for untreated and microwave plasma-treated samples for 30, 60, 80, and 100 s at different current densities. **(C)**: Peak current (I) at different scan rates for untreated and microwave plasma-treated samples for 30, 60, 80, and 100 s **(D)**: log I vs. log V plot for untreated and microwave plasma-treated samples for 30, 60, 80, and 100 s.

Where I/S is the current density (mA/cm^2^), and ∆t is the discharging time measured in seconds. Areal specific capacitance (C_A_) based on scan rate from 10 to 100 mV/s is shown in Figure 10A. It is observed that the specific capacitance shows decreasing behavior with the increase in scan rate. Here the untreated sample has the areal capacitance of approximately 15.4 mF/cm^2^ at 10 mV/s. Further increase in plasma treatment time as 30 s and 60 s, we observe a gradual increase in areal capacitance, approximate 18 mF/cm^2,^ and 20 mF/cm^2,^ respectively. As we reach the time at 80 s, the C_A_ values enhanced approximately up to 20.826 at 10 mV/s, as shown in Figure 10A. Further, at 100 s of microwave plasma treatment, TiO_2_ shows the lowest value of areal capacitance of 15.37 mF/cm^2^ at 10 mV/s near the untreated sample.

Similarly, the areal capacitance behavior is also determined from the GCD curve at a current density of 0.2–1.4 mA/cm^2,^ as shown in Figure 10B. For untreated sample the C_A_ approximate ∼225 mF/cm^2^ at 0.2 mA/cm^2^ current density. As the plasma treatment time increases, the areal capacitance becomes 85 mF/cm^2^, 81 mF/cm^2^, and 230 mF/cm^2^ for 30, 60, and 80 s respectively.

At 80 s microwave plasma treatment areal capacitance has maximum value which is approximate 24.59 mF/cm^2^ at 1 mA/cm^2^ current density and 230 mF/cm^2^ at 0.2 mA/cm^2^ current density. Further increasing plasma treatment time at 100 s, a decrement has been observed in areal capacitance up to 181 mF/cm^2^. Therefore, CV and GCD analysis observed that the electrochemical performance starts showing decreasing behaviors as we go further plasma treatment time beyond 80 s. Maximum anodic and cathodic current at different scan rates is shown in Figure 10C. With increasing the scan rate from 10 to 100 mV/s, the anodic peak current shifted towards a higher potential, and *vice versa* in the case of the cathodic peak for all samples. This nature of the electrode shows excellent reversibility. It also shows the 80 s having a higher current than untreated and other plasma-treated samples. According to the charge storage mechanism, capacitance is mainly divided into two categories: the first surface-controlled capacitance and the second is diffusion-controlled capacitance. The capacitive mechanism is recognized using relation I = a v^b^, where ‘I' is the current density, ' v' is the scan rate, and ‘a' and “b” are the constants. Figure 10D shows a plot between log(i) and log(v), by linear fitting of log(i) vs. log(v), b value calculated using power law expression such as: i = a. v^b^. From literature, if “b”∼0.5, capacitance controlled by diffusive process, but for b = 1, revealed the capacitive contribution. All untreated and treated samples have a ‘b' value nearly equal to 0.5. It means intercalation -diffusive mechanism dominates over capacitive action for charge storage.

Further, the contribution of each individually capacitive and diffusion-controlled capacitance in charge storage is determined using the below relation. Generally, total current at fixed voltage termed as i(V) is a combination of capacitive current and diffusion-controlled current termed as $K_{1}v$ and $K_{2}\surd v$ respectively shown in relation (Iqbal et al., 2020).

$$i\left(V\right)=K_{1}v+K_{2}\surd v$$

**FIGURE 11:**
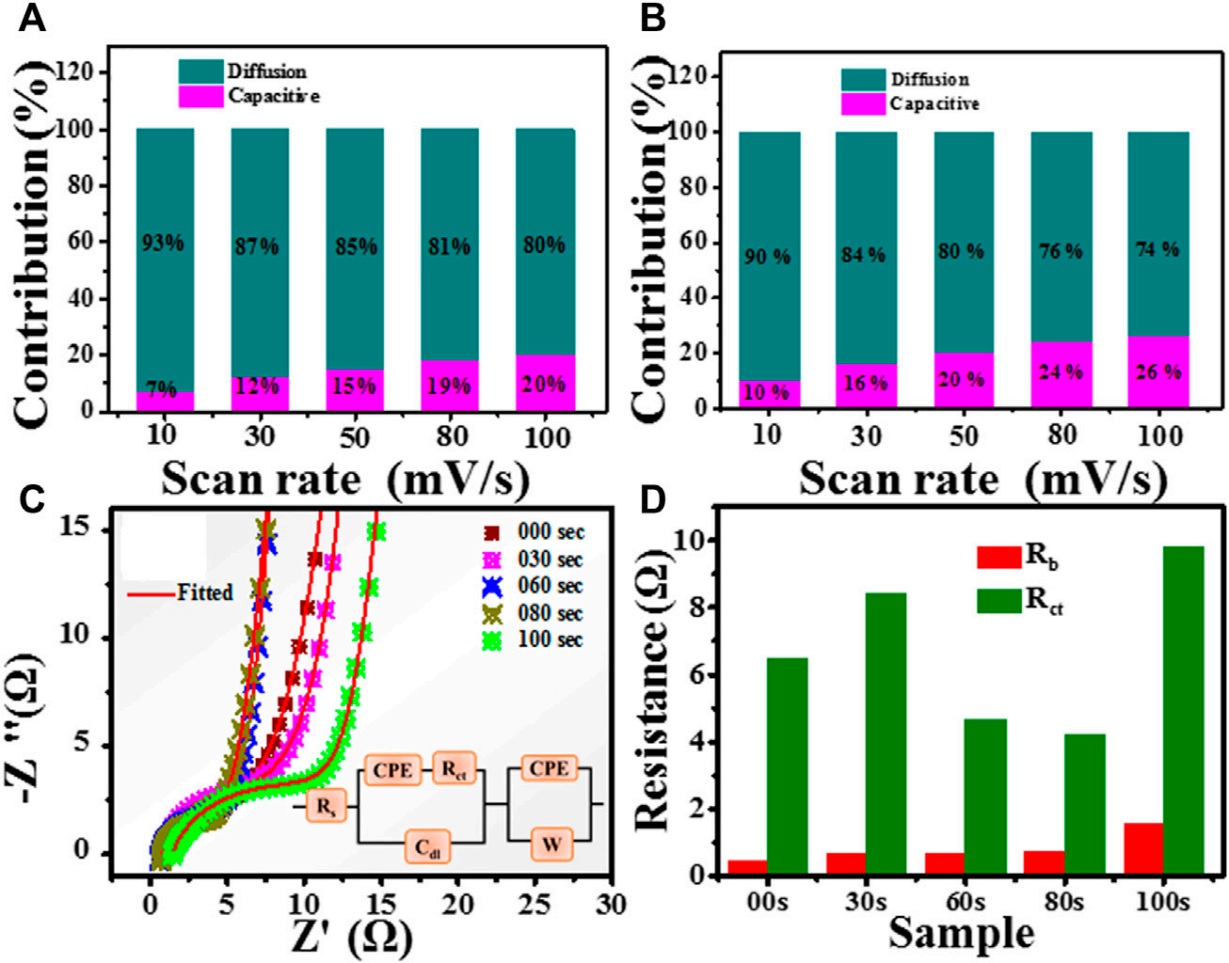
Comparison of capacitive contribution and diffusion-controlled contribution in **(A)** untreated **(B)** 80-s plasma treatment sample at different scan rates. **(C)**: Nyquist plot for untreated and treated samples. **(D)**: determined bulk and charge transfer resistance for untreated and plasma treated samples.

Where v is the scan rate (mV/s), K_1_ and K_2_ are constants. By linear fitting the plot between i/√v and √v , we calculated the K_1_ and K_2_ values. Using K_1_ and K_2_, one can find the amount of capacitive contribution and diffusion-controlled capacitance. The capacitive and diffusion contribution in untreated and 80-s microwave plasma treated samples is shown in Figures 11A,B at different scan rates. Untreated sample has % diffusion and capacitive contribution as 93 (7), 87 (12), 85 (15), 81 (19) and 80 (20) at 10, 30, 50, 80 and 100 mV/s scan rates. Similarly, 80 s plasma treated sample has % diffusion and capacitive contribution as 90 (10), 84 (16), 80 (20), 76 (24) and 74 (26) at 10, 30, 50, 80 and 100 mV/s scan rates. It shows the maximum amount of areal capacitance that occurred from the diffusion-controlled process for untreated and 80-s plasma-treated samples. This data is commensurate with the b value determined from power law expression.

Further, the electrochemical changes process is recognized by electrochemical impedance spectroscopy. Electrochemical impedance spectroscopy was performed at open circuit voltage with an AC amplitude of 0 mV in the frequency range from 0.01 Hz to 10^5^ Hz. Figure 11C represents the Nyquist plot of untreated and plasma-treated samples. The fitting of EIS spectrum was carried using demo version of ZSimpWin 3.20d software. The analogue fitted circuit shown in inset of Figure 11C. The main area of interest is high-frequency regions.

A non zero intercept of Nyquist plot on real axis shows the series bulk resistance (R_b_). It also found that the 80 s have semicircles with a minor diameter. Further, diameter of small arc shows charge transfer resistance (R_ct_) which include the charge movement on the surface of electrode. From the Nyquist plot, the bulk resistance (R_b_) is determined by the intercept value of the high-frequency region of the Nyquist plot to the *x*-axis. Figure 11D shows the bulk and charge transfer resistance. It is clear 80s sample has the lowest charge transfer resistance. These impedance results strongly resemble the CV and GCD analysis presented above. C_dl_ is the double layer capacitance. The parallel combination of R_ct_ and C_dl_ is responsible for the small semicircle features. A straight line represents the capacitive action on electrode. The constant phase element (CPE) and Warburg constant (W) in the fitted circuit shows the diffusion disturbance at interface.

The areal energy density and power density determined using the formula

$$E_{A}=\frac{C∆V^{2}}{2*\;3600}\;andP_{A}=\frac{E}{discharge\;time}\times 3600$$

where C is the areal-specific capacitance (mF/cm^2^), ∆V is a potential window (V), E_A_ is the areal energy density (mW. h/cm^2^), and P_A_ is the areal power density (mW/cm^2^). Further at 0.6, 1.0 and 1.4 mA/cm^2^ current densities; the calculated energy and power density has been shown in Table 1.

**TABLE 1:** The calculated energy and power density for current density are 0.6, 1.0, and 1.4 mA/cm^2^.

Sample (s)	E_A_ (µW.h/cm^2^)	E_A_ at P_A_	E_A_ at P_A_
	at P_A_ (µW/cm^2^)	At 1 mA/cm^2^	At 1.4 mA/cm^2^
	At 0.6 mA/cm^2^		
0	1.161 at 299.83	0.763 at 500.34	0.679 at 700.40
30	1.75 at 299.83	0.769 at 500.34	0.78 at 700.40
60	2.37 at 299.83	0.972 at 500.34	0.78 at 700.40
80	9.68 at 299.83	3.396 at 500.34	1.843 at 700.40
100	2.708 at 299.83	1.03 at 500.34	0.875 at 700.40

From the above discussion, we found that untreated samples have a higher degree of agglomeration, which limits their electrochemical performance. As we carried out plasma treatment on TiO_2_ with 30 s and 60 s, agglomeration decreased, and active sites were increased. As a result, C_sp_ is increased. At 80 s treated, the sample has the best combination of optimism roughness and a low degree of agglomeration, providing an excellent electrochemical performance. With further increase in plasma treatment time, the roughness and agglomeration are decreased as confirmed by XRD and amorphous percentage parameter; consequently, a fall in capacitance occurred. At last, we analyzed that plasma treatment synergistically affects the TiO_2_ nanoparticle and improves their electrochemical performance. Moreover, a long-lasting plasma treatment may destroy the electrochemical performance.

#### 3.5.1 Comparision with literature

Literature reveals that in the last few decades, much work has been performed on TiO_2_ at different prospective phases, including the doping and plasma treatment. Here, a few are discussed, and a comparison is carried out with the present work. Wu et al. (2013). published two reports in 2013 based on plasma treatment on TiO_2_ nanotube; the second is based on the hydrogenation method. They obtained highest capacitance approximate 7.22 mF cm^−2^ at 0.05 mA cm^−2^ current density and 20.08 mF cm^−2^ at a current density of 0.05 mA cm^−2^ (Wu et al., 2014) and power density is 13.50 mW cm^−2^ and 19.29 mW cm^−2^ respectively. In our study maximum, the specific capacitance value is 230 mF/cm^2^ at 0.2 mA/cm^2^ current density, which is ∼31 times higher than *Hui Wu et al.* reported value. Furthermore, our electrode shows the maximum highest power density of 500 μW/cm^2^ and the maximum energy density of 3.396 µWh/cm^2^ at 1 mA/cm^2^ current density. Yibing Xie et al. (2013) reported the TiO_2_ nanotube array and nanopore array-based supercapacitor. TiO_2_ nanopore have maximum capacitance 7.8 mF cm^−2^ at 20 mV/s. From GCD, the specific capacitance of the TiO_2_ nanopore array is determined to be 11.8 mF cm^−2^ at a current density of 0.2 mA/cm^2^. In our work, After 80-s plasma treatment, the C_A_ increased to 20.826 at 10 mV/s. Similarly, the areal capacitance determined from GCD curve having value is 230 mF/cm^2^ at 0.2 mA/cm^2^ current density. He Zhou and Zhang., 2013 reported that a facile cathodic reduction process modifies the synthesis of TiO2 nanotube arrays. Maximum areal specific capacitance of TiO_2_ is 0.949 mF/cm^2^ at 0.01 mA/cm^2^ (Zhou & Zhang, 2013). In our case, its value is 230 mF/cm^2^ at 0.2 mA/cm^2^ current density, which is ∼230 times higher than *He Zhou et al.* reported value. Qirong Ke et al., 2016 reported a three-dimensional TiO_2_/graphene porous composite for supercapacitor characteristics (Ke et al., 2016). The specific capacitance of the carbon/TiO_2_/reduced graphene oxide composite has been enhanced up to 23.6 mF/cm^2^ at 0.1 mA/cm^2^, which is ∼10 times lower than our obtained values. Jianfang Zhang et al., 2016 reported the supercapacitor behavior of highly ordered reduced TiO_2_ nanotube arrays (TNAs) (Zhang et al., 2017). They offer the highest specific capacitance of 23.24 mF cm^−2^ at a scan rate of 2 mV/s, which is ∼ nearly identical to our current results. Jinguang Cai et al., 2017 reported that carbon/TiO_2_ micro-supercapacitor has the highest specific capacitance up to 27.3 mF cm^−2^ at a current density of 0.05 mA cm^−2^. The capacitance from CV is 15.5 mF cm^−2^ at a typical scan rate of 10 mV s^−1^ (Cai et al., 2017). In our case, plasma-treated TiO2 has a specific capacitance of 20.826 at 10 mV/s, which is ∼34.4% higher. Jie Meng et al., 2018 reported that the effect of plasma treatment on graphene fiber under ambient conditions has the highest areal-specific capacitance, 36.25 mF/cm^2^ at 0.1 mA/cm^2^ current density (Meng et al., 2018), which is six times lower than that of our case. Priyanka Rani. 2020 reported the graphene oxide and titanium dioxide hybrid (GO-TiO_2_) nanocomposites to fabricate flexible solid-state supercapacitors (Rani et al., 2020). The optimum composition of GO-TiO_2_ nanocomposite (TG25) exhibits a high areal-specific capacitance of 73.43 mF/cm^2^ at a current density of 0.5 mA/cm^2^. The fabricated device shows high power density (3.5 mW/cm^2^), a high energy density (0.007 mW/cm^2^). In the present study, we have C_A_ 80 mF/cm^2^ at 0.5 mA/cm^2^ current density, which is ∼9% higher than the *Priyanka Rani et al.* reported value. Furthermore, in the present work maximum highest power density is 500 μW/cm^2,^ and the maximum energy density is 3.396 µWh/cm^2^ at 1 mA/cm^2^ current density. Mohammad Qorbani et al., 2019 reported Ti-rich anatase TiO_2_ material-based supercapacitor devices (Qorbani et al., 2019). The areal capacitance of each electrode is 3.8 mF cm^−2^ at 25 μA cm^−2^. This solid-state device offers a maximum power density of 0.925 mW cm^−2^. In the present work, plasma treated has the highest specific capacitance, 230 mF/cm^2^ at 0.2 mA/cm^2^ current density, which is ∼60 times higher than Mohammad Qorbani *et al.* reported value. Mehri Maghsoudi et al., 2021, reported the super capacitance performance of polyaniline/H-TiO_2_ material, and this electrode has a maximum specific capacitance of ∼5.6 mF/cm^2^ at 0.1 mA/cm^2^ current density (Khameneh Asl et al., 2021). The present study has a specific capacitance of 230 mF/cm^2^ at 0.2 mA/cm^2^, which is ∼40 times higher than that of *Mehri Maghsoudi et al.* reported value. Therefore, from the above discussion, it is concluded that the microwave Air plasma exposure of TiO_2_ significantly impacts the electrochemical properties. Also, as per literature, it enhances the electrochemical properties by approximately 30 percent compared to the till-reported data.

## 4 Electrochemical performance in positive potential window

**FIGURE 12:**
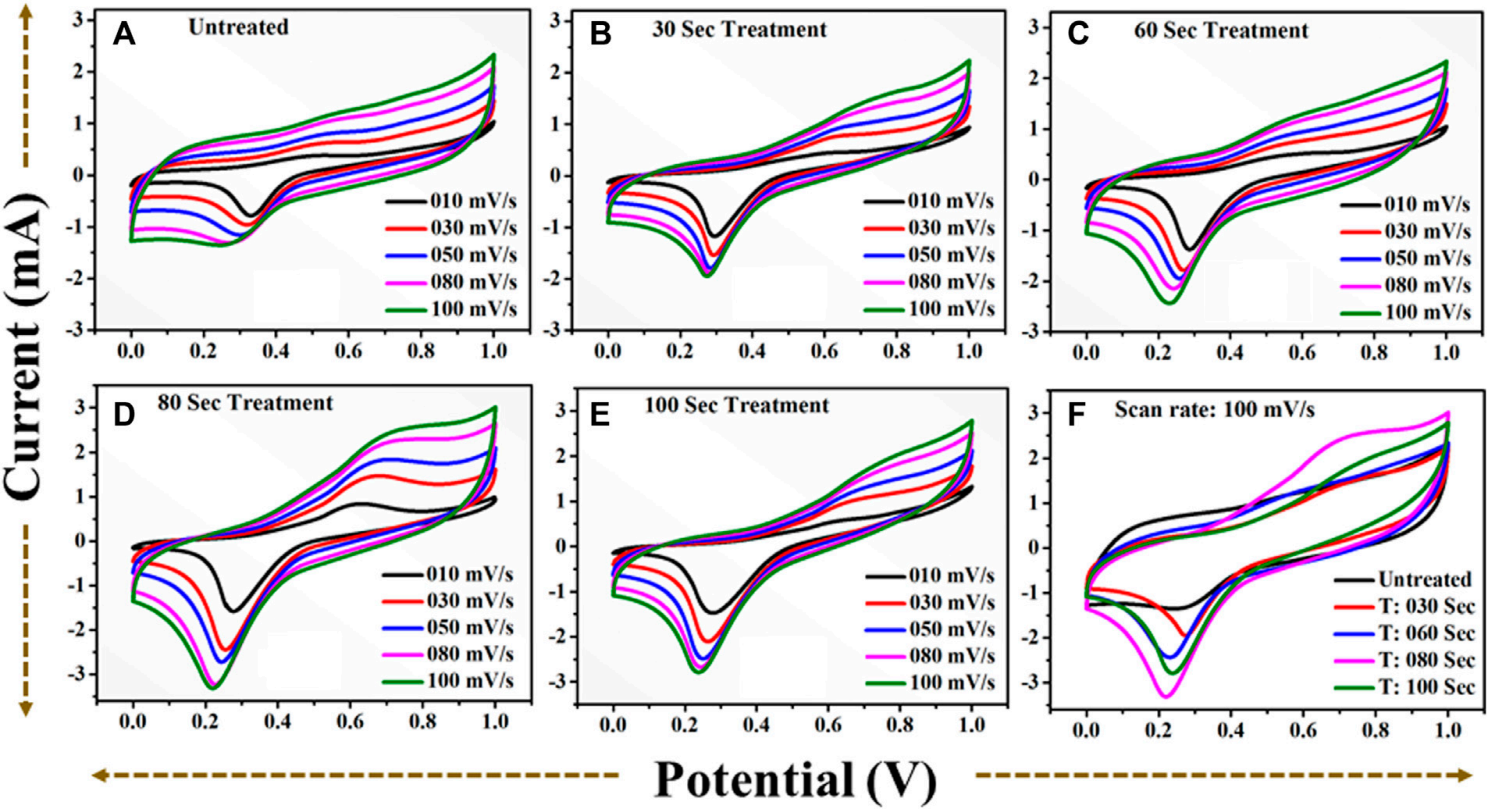
CV profile in positive potential window **(A)**: for untreated TiO_2_ at scan rate (10–100) mV/s. **(B)**: for 30 s plasma treated TiO_2_ at scan rate (10–100) mV/s. **(C)**: for 60 s plasma treated TiO_2_ at scan rate (10–100) mV/s. **(D)**: for 80 s plasma treated TiO_2_ at scan rate (10–100) mV/s. **(E)**: for 100 s plasma treated TiO_2_ at scan rate (10–100) mV/s. **(F)**: A comparative CV profile for **(A–E)** at scan rate 100) mV/s.

In order to verify the practical applicability of the plasma treated TiO_2_ based material as an electrode material for the supercapacitor application, we have carried out the CV and GCD measurement over the positive window. The CV profiles of the all the sample at different scan rate are displayed in Figure 12.

**FIGURE 13:**
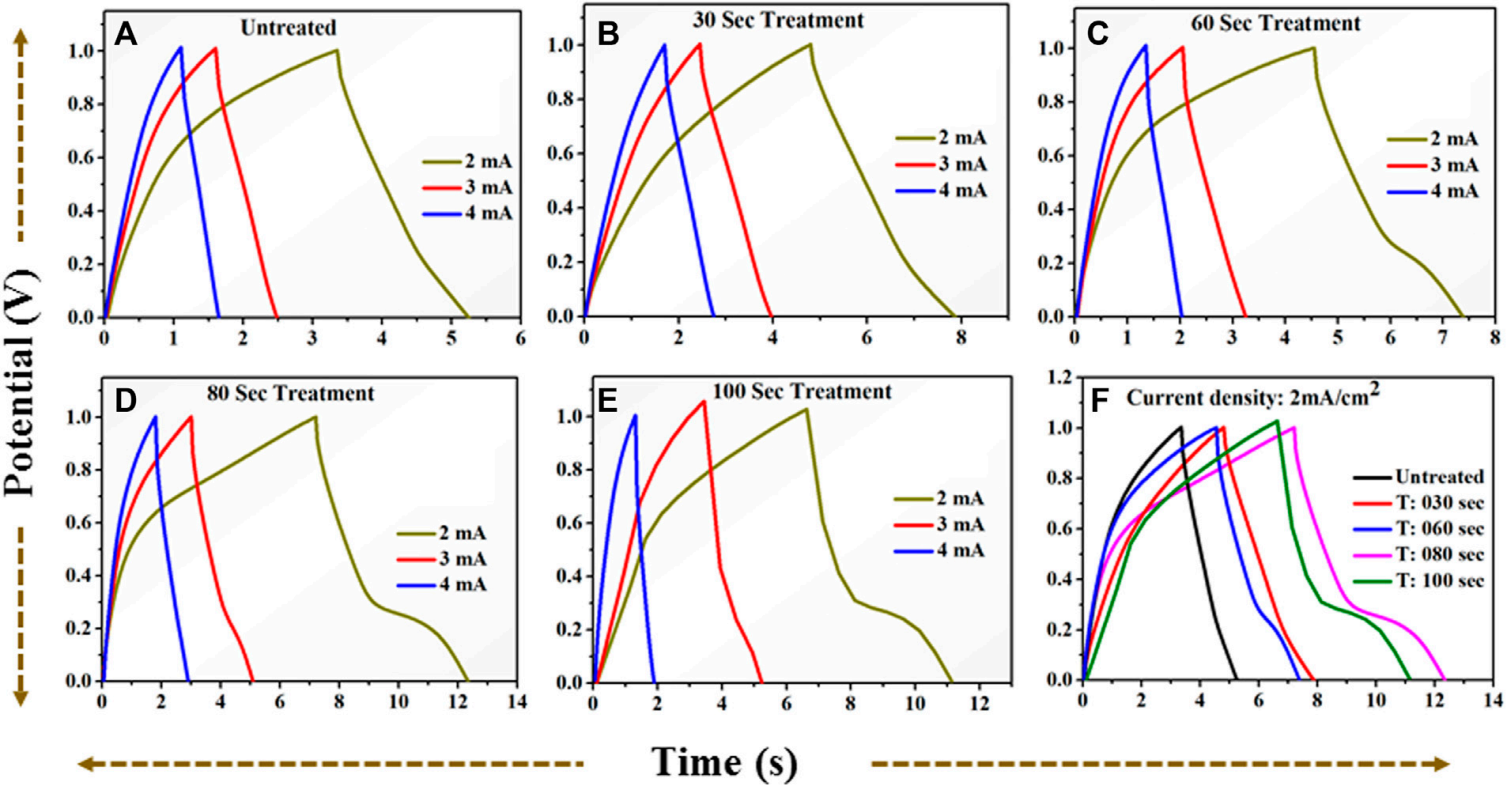
GCD profile for positive potential window **(A)**: for untreated TiO_2_ at current (2–4) mA. **(B)**: for 30 s plasma treated TiO_2_ at current (2–4) mA. **(C)**: for 60 s plasma treated TiO_2_ at current (2–4) mA. **(D)**: for 80 s plasma treated TiO_2_ at current (2–4) mA. **(E)**: for 100 s plasma treated TiO_2_ at current (2–4) mA. **(F)**: A comparative GCD profile for **(A–E)** at current 2 mA = 2 mA/cm^2^ current density.

**FIGURE 14:**
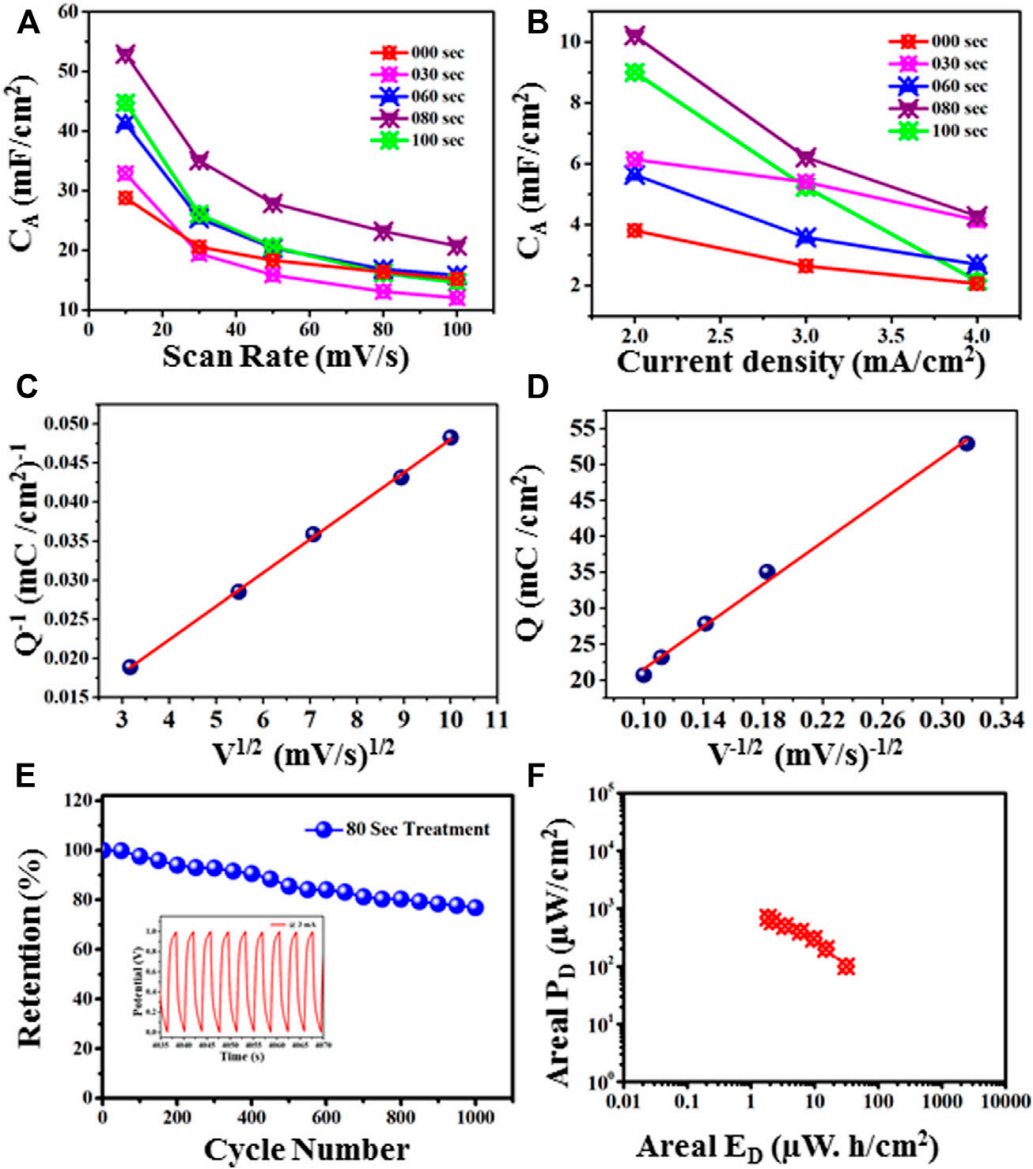
**(A)** Areal capacitance behaviour at different scan rates. **(B)**: Areal capacitance behaviour at different current densities. **(C)** Plot of Q^−1^ vs. V^1/2^ to find the total stored charge. **(D)** Plot of Q vs. V^−1/2^ to find the charge stored on the outer surface of the electrode material. **(E)** Cyclic stability at 3 mA, **(F)** Ragone plot of 80 s Treatment sample.

The area under the curve is expanding gradually with increase in scan rate demonstrating excellent performance of the all samples. One can notice that the 80 s plasma treated sample shows the large area under the curve revealing that the charge storage capacity of the electrode material has been significantly increased. The obvious redox peak in the CV curve of the all samples is observed indicating that the charges may be stored through both faradic and non-faradic processes. The specific capacitance values computed at the various scan rate shows that 80 s plasma treated sample exhibits overall higher values than other samples (Figure 14A). The similar observation has been observed for the CV profiles recorded in potential window of −0.5 to 0.5 V. The charge storage capability of the untreated and plasma treated samples is further investigated by GCD technique. The GCD profiles of the all samples obtained with different current densities are shown in Figure 13.

It is observed that the discharging time decreases with increasing current density. All GCD profiles shows the quasi-symmetric behaviour. The specific capacitance variation at different densities is plotted in Figure 14B. The 80 s plasma treated sample exhibits highest specific capacitance (10.2 mF/cm^2^ at 2 mA/cm^2^ current density) than their counterpart which may be due to the little amorphous nature induced due to the optimal plasma treatment. Furthermore, the plasma induced lattice expansion could provide the pathways for the diffusion of the charges inside the crystal structure which might enhances the charge storage ability of 80 s plasma treated sample.

Further, total charge, and charge of the outer, and inner surfaces of the 80-s plasma treatment sample has been calculated. Figure 14C shows the plot of calculated specific capacity (Q) against square roots of scan rates (V). The *y*-intercept of Figure 14C gives the total charge stored (Q_T_) by the electrode material (Isacfranklin et al., 2021). It follows below equation:

$$\frac{1}{Q}=constant*v^{1/2}+\frac{1}{Q_{T}}$$

The estimated total stored charge is 188 mC/cm^2^. Further, Figure 14D shows the correlation between calculated specific capacity (Q) and the inverse of scan rates. The *y*-intercept determines the charge stored at the outer surface (Q_O_) (Deshmukh et al., 2018). This plot follows this equation:

$$Q=constant*{v^{-}}^{1/2}+Q_{O}$$

The estimated charge stored was found to be 6.83 mC/cm^2^ and the charge stored on the inner surface is 181.85 mC/cm^2^. Moreover, in Figure 14E, the cyclic stability measurement of 80 Sec plasma treatment sample has been investigated using GCD at 3 mA current. It has been found that capacitance retention is 76.9% after 1,000 cycles. Figure 14F shows the Ragone plot of 80 sec plasma treatment sample.

## 5 Conclusion

We investigated the effect of microwave air plasma treatment on TiO_2_ pellets for 30 s, 60 s, 80 s, and 100 s in this study. The UV-visible research revealed that the band gap decreases as we approach the plasma treatment time of 80 s; however, if we continue with plasma treatment, the band gap begins to widen. FTIR studies reveal broad O-Ti-O vibrational bands, indicating the high disorder in the sample from the 80 s. Finally, electrochemical studies validate the effect of UV-visible, FTIR, and XRD for an 80-s plasma treatment time. Because one sample has a larger integral area under the curve than others. The electrode-potential drop (IR drop) for the 0 s, 30 s, 60 s, 80 s, and 100 s treated samples was 171 mV, 181 mV, 162 mV, 104 mV, and 154 mV, respectively, at 1 mA/cm two current density. For 80 s, the specific capacitance is increased to approximately 20.826 at 10 mV/s. Furthermore, after 100 s of microwave plasma treatment, TiO_2_ has the lowest areal capacitance of 15.37 mF/cm 2 at 10 mV/s, comparable to the untreated sample. Also, the electrochemical study in the positive potential window validates the 80-s plasma treatment sample possess excellent performance over other samples.

## Data Availability

The original contributions presented in the study are included in the article/Supplementary Material, further inquiries can be directed to the corresponding author.
