# Supplementary figures and images for: Enhancing the electrochemical performance of TiO2 based material using microwave air plasma treatment with an ECR cavity
Source: Front Chem. 2022 Nov 24;10:1065153. doi: 10.3389/fchem.2022.1065153 (PMC9729354; doi:10.3389/fchem.2022.1065153)

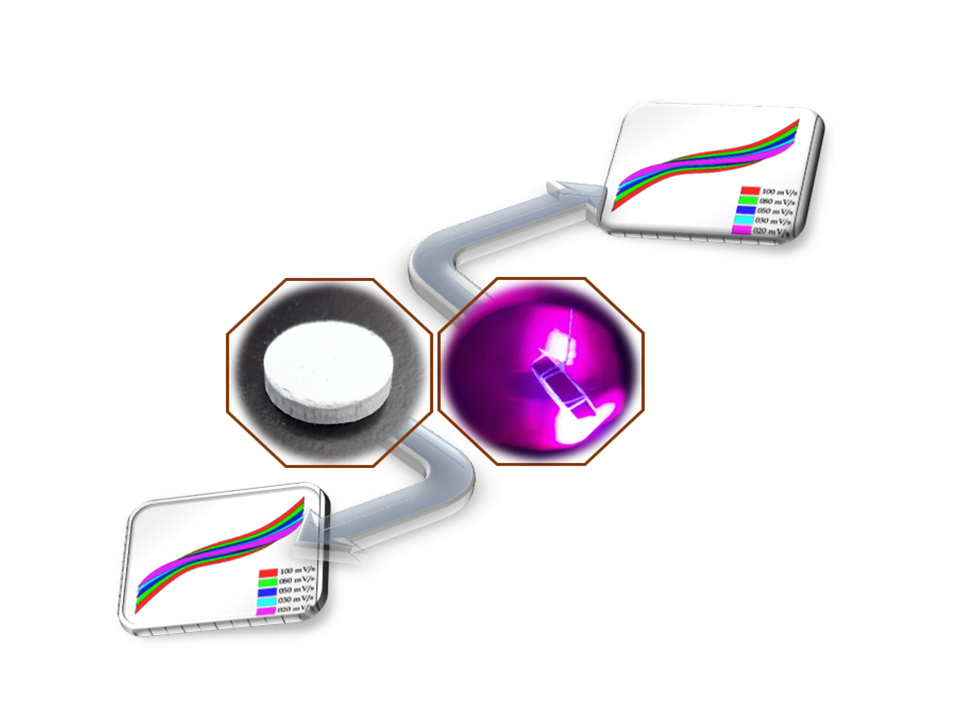

Supplement: Supplementary file 1 [file Image1.TIF]
